# Unusual morphology of foveal Müller glia in an adult human born pre-term

**DOI:** 10.3389/fncel.2024.1409405

**Published:** 2024-06-27

**Authors:** Deepayan Kar, Ramya Singireddy, Yeon Jin Kim, Orin Packer, Richard Schalek, Dongfeng Cao, Kenneth R. Sloan, Andreas Pollreisz, Dennis M. Dacey, Christine A. Curcio

**Affiliations:** ^1^Department of Ophthalmology and Visual Sciences, Heersink School of Medicine, University of Alabama at Birmingham, Birmingham, AL, United States; ^2^Department of Biological Structure, University of Washington, Seattle, WA, United States; ^3^Department of Molecular and Cellular Biology and Center for Brain Science, Harvard University, Cambridge, MA, United States; ^4^Department of Ophthalmology, Medical University of Vienna, Vienna, Austria

**Keywords:** fovea, Müller glia, preterm adult, volume electron microscopy, human

## Abstract

The fovea of the human retina, a specialization for acute and color vision, features a high concentration of cone photoreceptors. A pit on the inner retinal aspect is created by the centrifugal migration of post-receptoral neurons. Foveal cells are specified early in fetal life, but the fovea reaches its final configuration postnatally. Pre-term birth retards migration resulting in a small pit, a small avascular zone, and nearly continuous inner retinal layers. To explore the involvement of Müller glia, we used serial-section electron microscopic reconstructions to examine the morphology and neural contacts of Müller glia contacting a single foveal cone in a 28-year-old male organ donor born at 28 weeks of gestation. A small non-descript foveal avascular zone contained massed glial processes that included a novel class of ‘inner’ Müller glia. Similar to classic ‘outer’ Müller glia that span the retina, inner Müller glia have bodies in the inner nuclear layer (INL). These cells are densely packed with intermediate filaments and insert processes between neurons. Unlike ‘outer’ Müller glia, ‘inner’ Müller glia do not reach the external limiting membrane but instead terminate at the outer plexiform layer. One completely reconstructed inner cell ensheathed cone pedicles and a cone-driven circuit of midget bipolar and ganglion cells. Inner Müller glia outnumber foveal cones by 1.8-fold in the outer nuclear layer (221,448 vs. 123,026 cells/mm^2^). Cell bodies of inner Müller glia outnumber those of outer Müller glia by 1.7-fold in the INL (41,872 vs. 24,631 cells/ mm^2^). Müller glia account for 95 and 80% of the volume of the foveal floor and Henle fiber layer, respectively. Determining whether inner cells are anomalies solely resulting from retarded lateral migration of inner retinal neurons in pre-term birth requires further research.

## Introduction

The fovea is a specialized retinal feature of humans and most non-human primates that enables high-acuity and color vision. This region is characterized by densely packed cone photoreceptors within a foveal avascular zone (FAZ) and an excavation of inner retinal neurons, creating a shallow pit that is visible in the ocular fundus (Provis et al., 2013; Bringmann et al., 2018). Similar to non-foveate species, human retinal development exhibits overlapping fetal epochs of retinogenesis, neurogenesis, and synaptogenesis (Cepko, 2014; Hoshino et al., 2017; Zhang et al., 2020). Foveal neurons that express key molecular markers earlier than peripheral neurons are still immature at birth (Hoshino et al., 2017). Crowding of cone inner segments into the foveal center, together with migration of inner retinal neurons out of the pit, is a remarkable process starting late in fetal life and extending beyond birth (Hendrickson, 2005; Hendrickson et al., 2012). Thus, foveal structure and function are susceptible to perturbation by pre-term birth.

Foveal maturity can be assessed in clinic populations with optical coherence tomography (OCT), a depth-resolving imaging method that reveals layers of retina and choroid (Hendrickson et al., 2012; Thomas et al., 2020). Due to extended foveal development, a neonate born at term may exhibit inner retinal layers deployed across the pit and fewer than normal outer retinal reflective bands representing photoreceptor and retinal pigment epithelium (RPE) (Maldonado et al., 2011; Dubis et al., 2012). Imaging hallmarks of a premature birth include small FAZ, small pit volume, and at the foveal center, thickened inner retinal layers relative to the outer nuclear layer (Mintz-Hittner et al., 1999; Bowl et al., 2016, 2018b; Sjöstrand and Popovic, 2020). This constellation has been called macular (Bowl et al., 2016) or foveal (Sjöstrand et al., 2017) developmental arrest and occurs independently of a more severe condition, called retinopathy of prematurity (Mangalesh et al., 2022). Arrest is accompanied by reductions in visual acuity, photopic sensitivity, and electroretinographic response (Fulton et al., 2005; Akerblom et al., 2016; Bowl et al., 2016; Altschwager et al., 2017).

While neurons and associated glial cells migrate away from the pit, a small population of glia, potentially significant, remains at the pit center. Classic Müller glia have somata in the inner nuclear layer, spanning from internal to external limiting membrane, and intercalating among neurons to provide diverse services such as structural support, water homeostasis, retinoid recycling, and sequestration of a characteristic yellow pigment (xanthophyll carotenoid) (Wang and Kefalov, 2009; Daruich et al., 2017; Bringmann et al., 2018; Pollreisz et al., 2020). An inverted cone-like plug of glial tissue on the foveal floor was observed in early electron microscopy studies (Yamada, 1969). Named a “Müller cell cone,” this feature was considered by ophthalmologists as a repository for xanthophyll pigments and a participant in tractional disorders of the inner retinal surface (Gass, 1999). Accordingly, in a single-section transmission electron microscopy of one human and one macaque fovea, Syrbe et al. described a population of 25–35 large glial cell bodies in the foveal center that seemed to have little contact with adjacent neurons (Syrbe et al., 2017; Bringmann et al., 2018). How such specialized glia might participate in foveal development has not been studied.

With the long-term goal of characterizing the human fovea at the nanoscale, we acquired retinal tissue from an adult who was born pre-term, exhibiting features of arrested development. To document Müller glial morphology, abundance, and neural contacts in this fovea, we 3-dimensionally reconstructed glia using volume electron microscopy. This technique permits an unprecedented view of these elaborate, large, and numerous cells and builds on seminal two-decade-old studies of macaque retina that reconstructed glia manually (Burris et al., 2002; Ahmad et al., 2003). Volume electron microscopy has been recently used to detail neural circuitry and development of the fovea-abundant midget system for color and acute vision (Wool et al., 2019; Zhang et al., 2020). Herein, we identify a non-canonical glial phenotype, localized entirely within the inner retina, with cell bodies in the inner nuclear layer. These cells may be a heretofore unrecognized component of the incomplete inner retinal migration of prematurity.

## Materials and methods

### Compliance

Tissue was collected in collaboration with eye bank and organ procurement organizations (SightLife and Northwest LifeCenter, Bellevue, WA) and complied with the regulations of the respective agencies. The study was approved by the institutional review board at the University of Washington and the University of Alabama at Birmingham. All research activities adhered to the tenets of the Declaration of Helsinki and complied with the Health Insurance Portability and Accountability Act.

### Overview of study design

From one tissue volume of a human fovea of an adult born pre-term, we reconstructed in their entirety two Müller glia contacting the central-most cone. An outer cell spanned the retina and an inner cell spanned from the inner limiting membrane (ILM) to the outer plexiform layer (OPL). The central-most cone was identified by the qualitatively high packing density of cones just internal to the external limiting membrane (ELM). Additional analyses determined the number of cell bodies belonging to Müller glia and cones in the inner and outer nuclear layers (INL, ONL) and volume occupancy of neurons and glia in different layers.

### Donor characteristics

Donor eyes from a 28-year-old male born pre-term at 28 weeks of gestation due to placental abruption, per family report, were analyzed. This gestational age is considered very pre-term by the international clinical consensus (Karnati et al., 2020).

### Tissue preparation

Whole globes were recovered from the donor at the termination of artificial life support for organ recovery. Tissue was preserved as described (Wool et al., 2019; Zhang et al., 2020). Enucleated eyes were transected at the limbus, drained of vitreous, and placed in warm oxygenated Ames’ culture medium for 2 h. The retinas were maintained in this medium at 37°C and carefully dissected from the sclera before preservation. After isolation, the central retina (including the fovea) was dissected and placed in a 4% glutaraldehyde solution for approximately 2 h of fixation. The foveal tissue was then rinsed thoroughly in cacodylate buffer (0.1 M, pH 7.4) and incubated in a 1.5% potassium ferrocyanide and 2% osmium tetroxide (OsO_4_) solution in 0.1 M cacodylate buffer for 1 h. After washing, the tissue was placed in a freshly made thiocarbohydrazide solution (0.1 g thiocarbohydrazide in 10 mL double-distilled H_2_O heated to 600°C for 1 h) for 20 min at room temperature (RT). After another rinse at RT, the tissue was incubated in 2% OsO_4_ for 30 min at RT. The samples were rinsed again and stained *en bloc* in 1% uranyl acetate overnight at 40°C and washed and stained with Walton’s lead aspartate for 30 min. After a final wash, the retinal pieces were dehydrated in a graded alcohol series and placed in propylene oxide at RT for 10 min. The tissue was then embedded in epoxy resin (Durcupan). Semi-thin vertical sections through the retinal layers (0.5–1 μm thick) were cut and stained with toluidine blue and examined to determine the location of the foveal center.

### Sectioning and imaging

From a region approximately 250 μm on a side, surrounding the foveal center from the level of the cone outer fibers to the internal limiting membrane (ILM), ~4,500 80-nm serial tangential sections were imaged using automated tape ultramicrotomy scanning electron microscopy (Tapia et al., 2012; Hayworth et al., 2014; Kasthuri et al., 2015). Tape sections were imaged using a scanning electron microscope (Zeiss) (Kasthuri et al., 2015), generating an image volume with a voxel resolution of 5 × 5 × 60 nm in X, Y, and Z, respectively. Images were acquired in two separate sessions, 2015 for the outer retina and 2017 for the inner retina. The small foveal pit (2017) prompted an investigation into eye bank records and the discovery of the pre-term birth in a family interview.

### Cellular and sub-cellular reconstruction

TrakEM2 plug-in of the Fiji framework (ImageJ, National Institutes of Health, Bethesda, MD) was used to create a digital library consisting of tangential EM sections and navigate and descriptively analyze the multi-terabyte tissue volume (Cardona et al., 2012). Tangential sections were aligned, and cells were manually annotated using a pen display (Cocks et al., 2018; Cintiq 22HDT, Wacom, Kazo, Japan). The two separate imaging sessions produced two volumes. The two sets of annotations were aligned using manually placed landmarks in sections near the common border. Highly resampled three-dimensional (3D) volumes of individual cells were rendered and imported to a 3D visualization, processing, and analysis software (Dragonfly v4.1, Object Research Systems, Montréal, Canada). Using non-orthographic projection, specific features of 3D reconstructions were highlighted to establish neural-glial relationships. Landmarks in videos were annotated (Premiere Pro, v13.1, Adobe Inc., San Jose, CA) and exported in video format (H.264, mp4, 30 fps, high bitrate).

A custom program developed in MATLAB R2018a (MathWorks Inc., Natick, MA) normalized differences between horizontal sections using a reference histogram propagated to each section in the EM stack. A radial section through the fovea was orthogonally extracted from the dense stack of tangential serial sections. Due to the high density (16 tangential sections/ *μ*m) and precise alignment of the sections in the stack, a high-resolution reconstruction of orthogonal radial sections was achievable.

### Identifying Müller populations

Rapid tissue recovery after cessation of life-support coupled with immediate oxygenation before fixation yielded exceptional retinal fine structure, which is evident from the excellent preservation of cellular membranes and intracellular architecture. Identification of Müller glia was based on the presence of numerous electron-dense microfilaments in the cytoplasm (Krebs and Krebs, 1991; Syrbe et al., 2017) and a cell body in the inner nuclear layer (INL) which could be traced to process in adjoining layers. Müller cell nuclei were found to be more spherical than the nuclei of adjacent INL neurons.

Skeletonized individual Müller glia were classified as outer (classical) or inner (novel) based on their vertical extension through the neurosensory retina. The outer cells extended from ILM to ELM and spanned the entire tissue block. The inner cells did not extend below the OPL.

### Cell counts and spatial distribution of Müller cells

Quantitative measures of interest were the number of Müller glia contacting each cone’s outer fiber, cone:Müller ratio, and inner:outer Müller ratio. Using 25.6 × 15.9 μm fovea-centric, tangential sections in the INL and ONL, annotated locations of Müller cells and neurons were exported for morphometric analysis (Burris et al., 2002; Ahmad et al., 2003). Individual cells were identified through serial sections. To count inner and outer Müller cells, INL nuclei were identified in the dense EM volume, and the vertical and lateral extents of each cell were subsequently skeletonized to aid classification. Cone outer fibers and outer Müller trunks in the ONL and counts of the two Müller subtypes in the INL were determined and used for derived parameters. For unbiased sampling, cells overlapping two adjacent edges and intervening corners of the counting area were excluded. To show spatial distributions in the INL and ONL, centers of identified cells within a bounding box were plotted (Systat Software, San Jose, CA).

### Müller glia biomass

Müller glial abundance was expressed as a volume fraction in each retinal layer calculated by the percentage of area covered by glia. Area was computed in four 15 × 15 μm randomly chosen samples at similar distances from an empirically defined central-most foveal cone (index cone).

### Chromogenic immunohistochemistry of macula-wide cryosections

Ultrastructural findings were compared to immunoreactivity of glial fibrillary acidic protein, (GFAP) a marker for astrocytes and Müller glia in the human retina (Bringmann et al., 2018). Whole eyes were obtained from deceased human donors to the Advancing Sight Network (Birmingham AL, United States). Criteria for acceptance were as follows: ≥80 years of age, white, non-diabetic, and ≤ 6 h death-to-preservation. Eyes were typically preserved (in 4% buffered paraformaldehyde) between 3.5, and 6 h after death. They were screened for pathology using *ex vivo* multimodal imaging (Messinger et al., 2023). Nine eyes with unremarkable central retinas were used for this study. Detailed methods based on our previous studies (Cao et al., 2021; Chen et al., 2022; Anderson et al., 2023) are shown in Supplementary materials.

## Results

### Donor characteristics and foveal anatomy

**Figure 1:**
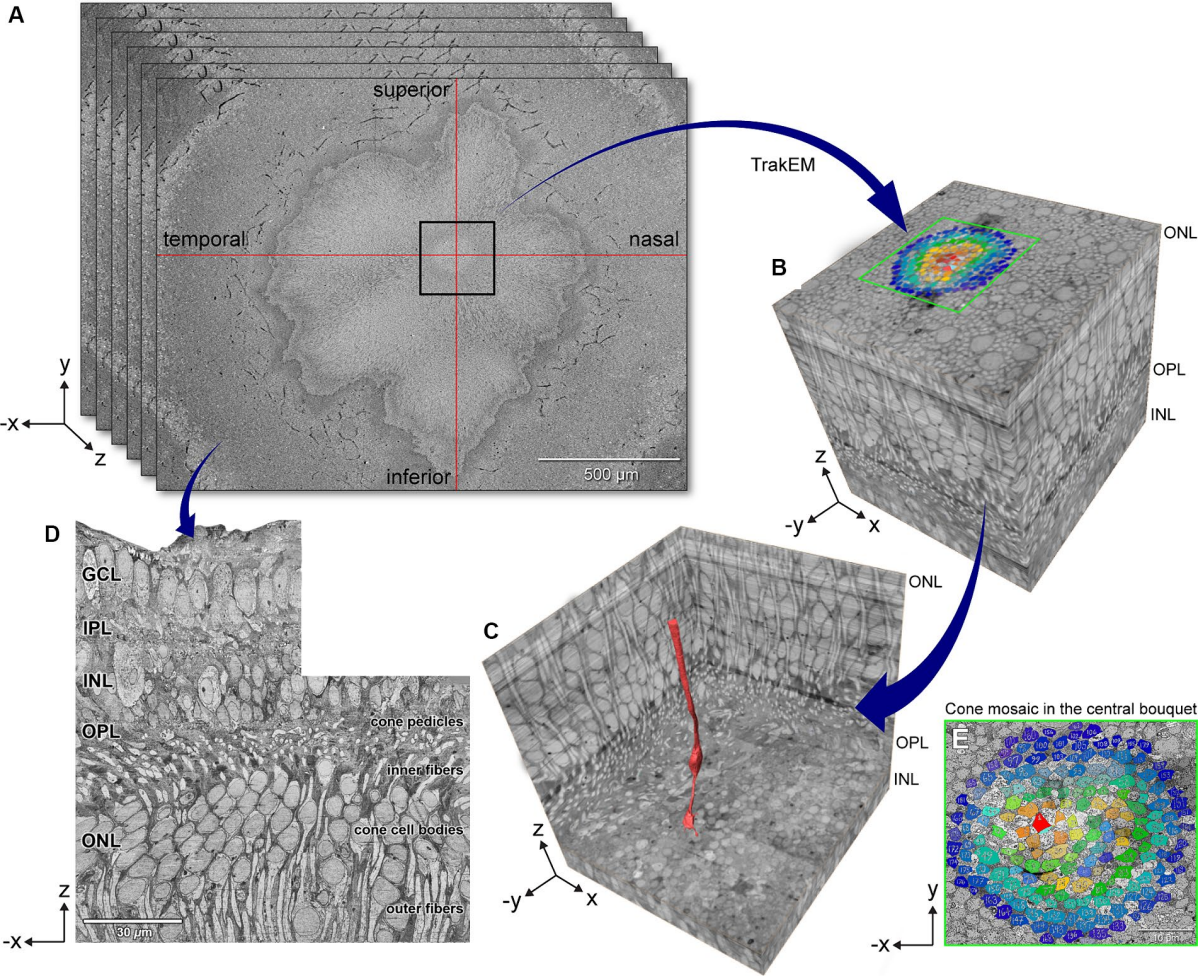
Volume electron microscopy technique for exploring foveal connectomics. **(A)** 4,500 80-nm serial horizontal sections were imaged using automated tape ultramicrotomy scanning electron microscopy and registered to create a dense stack of 16 sections/μm. **(B)** 3D volume shows the eccentricity-dependent color-coded cone mosaic in the central foveal cone bouquet (Panel E). **(C)** The central-most cone (red) is shown in context within the cone array. **(D)** Orthogonally reconstructed radial section was reconstructed from a dense stack of horizontal sections that underwent contrast normalization. GCL, ganglion cell layer; IPL, inner plexiform layer; INL, inner nuclear layer; OPL, outer plexiform layer; ONL, outer nuclear layer. Scale bars: panel A = 500 μm, panel D = 30 μm.

We analyzed the eye of a white 28-year-old male organ donor who was born pre-term at 28 weeks of gestation and who had a medical history of autism, per parent interview with eye bank personnel. Figures 1A,B shows how 4,500 60-nm serial tangential sections in a region 250 μm on a side surrounding the foveal central bouquet were horizontally sectioned (parallel to retinal layers) using an automated tape ultramicrotome. Sectioning was terminated before the external limiting membrane (ELM), as shown in a single reconstructed vertical slice (Figure 1D). As shown in Figures 1C,E, the location of the highest subjective density of cone outer fibers (between the cell body and the ELM) was designated as the foveal center. Within this region, a cone at the center (red, Figure 1C) was designated the index cone to guide subsequent exploration.

**Figure 2:**
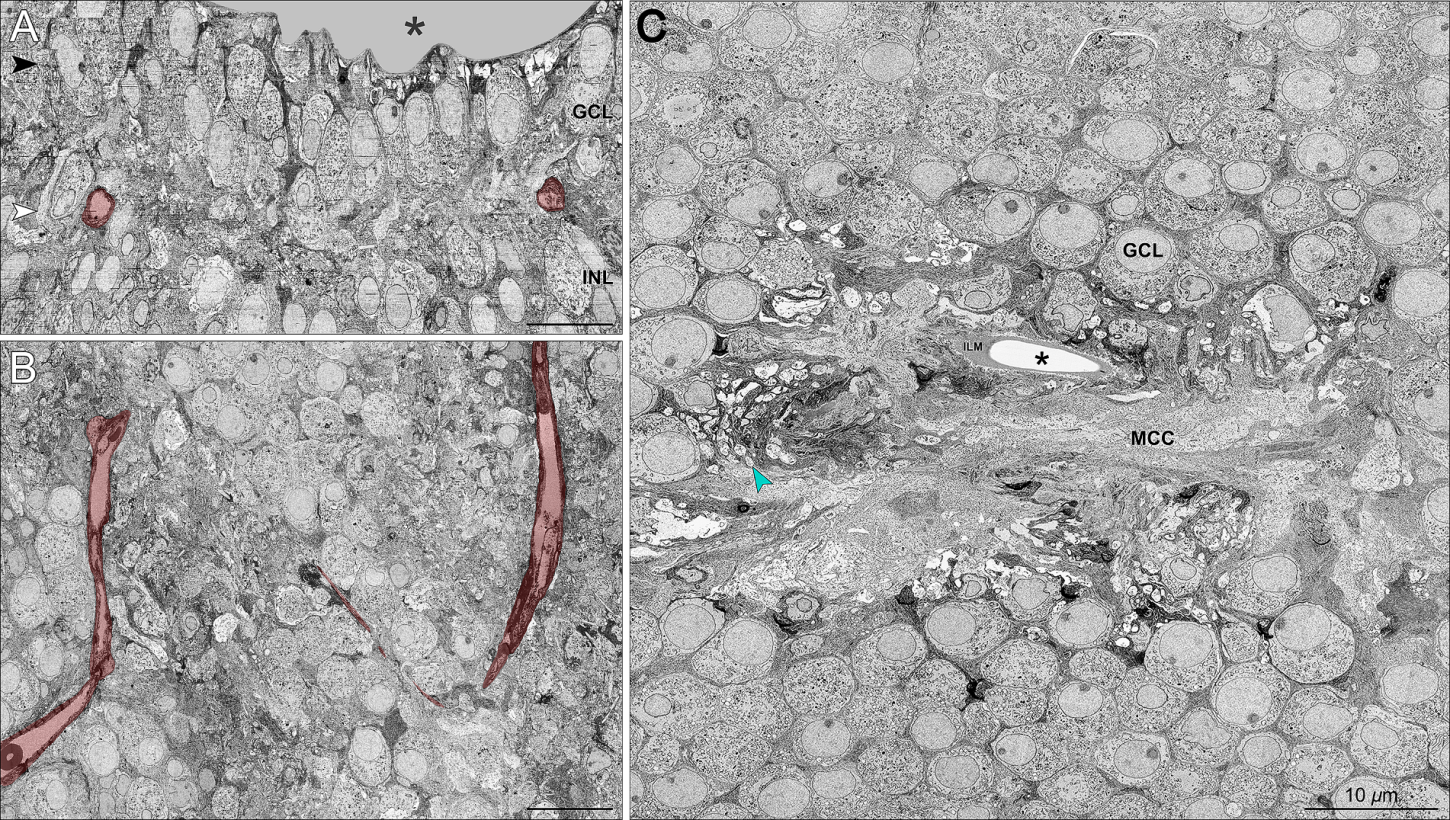
‘Müller cell cone’ and retinal neurons in the foveal avascular zone. **(A)** Vertical section sampled from the foveal center shows the foveal pit (*asterisk*) and the presence of GCL and INL neurons in relationship to the foveal vasculature labeled in red. A wider view of the foveal floor, at a lower magnification, is shown in Figure 1D. **(B)** Horizontally oriented electron micrograph of the GCL corresponding to the *white arrowhead in Panel A* shows a terminal capillary net forming a non-descript foveal avascular zone. **(C)** Horizontal section through the GCL (corresponding to the *black arrowhead in Panel A*) demonstrates the conglomeration of numerous glial processes forming a glia-enriched mass considered a ‘Müller cell cone’ (MCC) in the central foveal pit, i.e., umbo (asterisk). The MCC is surrounded by numerous mitochondria-rich retinal ganglion cell bodies and axons (green arrowhead). ILM, internal limiting membrane. Scale bars = 10 μm.

Figure 2 supports the family report of pre-term birth by showing that the center of the foveal depression (umbo) in these horizontally oriented sections was abnormal. Rather than a pit, there was a small dip shaped like a slit. This, in turn, was lined by the ILM and a mass of glial processes. This mass was similar to that described recently in humans and monkeys (Syrbe et al., 2017) and equated to the “Müller cell cone” in humans (Yamada, 1969, Hogan et al., 1971, Hendrickson et al., 2012; Figure 2C). Reassembly of horizontal sections for viewing in the vertical plane (Figures 2A,B) showed a small (50 μm diameter) non-descript foveal avascular zone. Unlike fully excavated foveal depressions, the foveal floor of this eye was lined by residual inner retinal layers, i.e., GCL, IPL, and INL, which were thinned. External to the inner retinal layers are the OPL with a row of cone pedicles that large, dispersed, and connected by long telodendria, much like those found in the peripheral retina. Of note, these structures had been investigated in detail prior to the inner retinal studies, without raising suspicions of abnormality, as pedicles were of expected structure and dimensions. Supplementary Video S1 shows a vertical melt-through of many horizontal sections. Below the pedicles (Supplementary Video S1) is a thin HFL in which the distal end of cone axons spread laterally, still interleaved by Müller glia. Within the foveal ONL on Supplementary Video S1, numerous cone cell bodies are tiered. The outer part of the foveal ONL is comprised of outer fibers of the cone photoreceptors, still interleaved with Müller glia outer trunks. These were all strictly parallel, i.e., no crossing of fibers.

**Figure 3:**
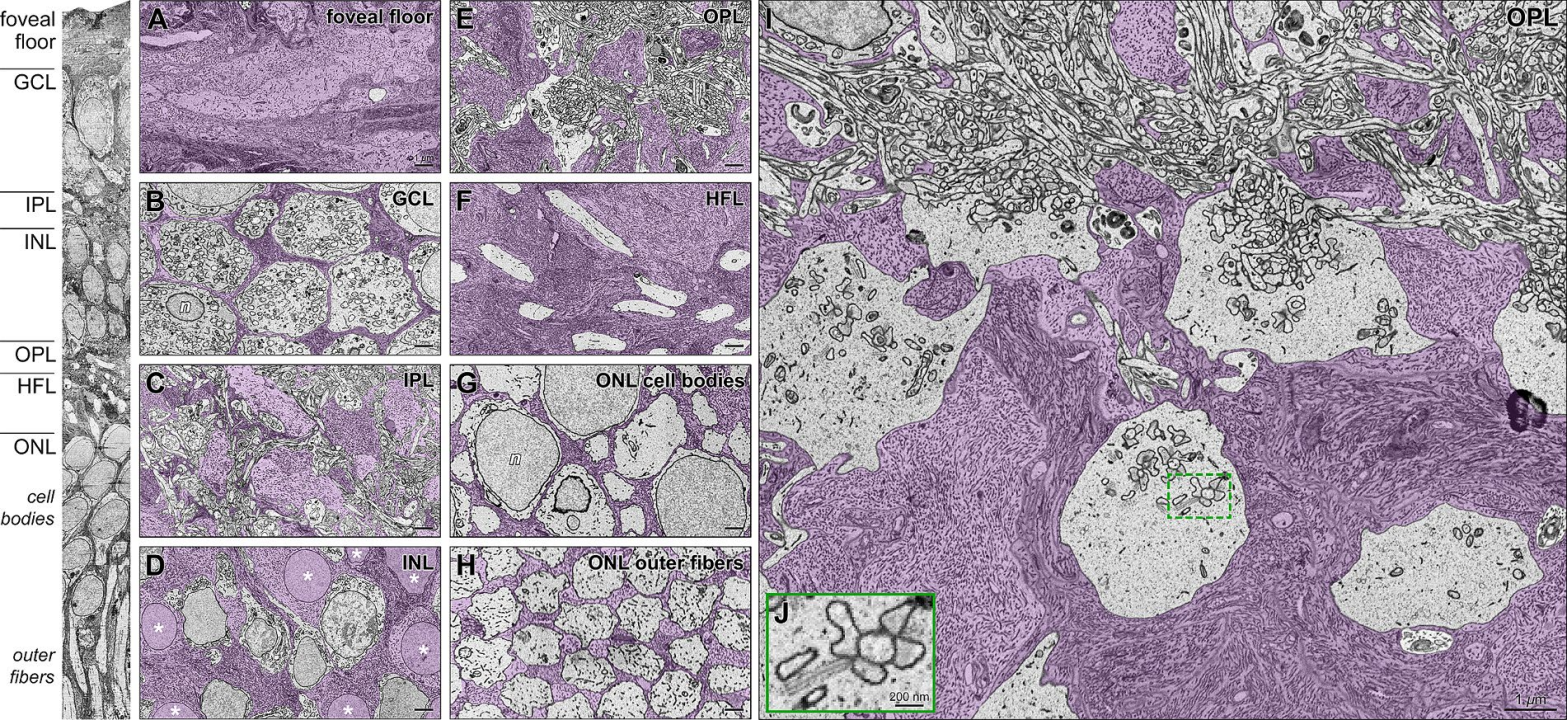
Foveal Müller glia and neurons in individual retinal layers, including cone pedicles in the outer plexiform layer. High-resolution transmission electron microscopy (TEM) of horizontal sections (Panels A-I) corresponding to layers indicated in the vertical slice (left). Müller glia in purple are densely filled with intermediate microfilaments. **(A)** Foveal floor demonstrates extensive conglomeration of Müller glial processes. **(B)** Thin glial processes wrap around the ganglion cell bodies. **(C)** Complex glial columns traverse through the neuropil of the INL and form smaller processes around dendrites in the IPL. **(D)** Somas of Müller glia demonstrate (asterisks) smooth nuclear membranes surrounded by voluminous intermediate filament-rich cytoplasm. They are surrounded by cell bodies of bipolar and horizontal interneurons. **(E)** In the OPL, intricate glia processes surround elements of the neurophil and wrap pedicles (cone photoreceptor terminals; details in Panel I). **(F)** The HFL is densely packed with large Müller processes interleaved with cone photoreceptor axons. **(G)** In the ONL, multiple Müller cell processes wrap around each cone cell body. **(H)** Also, in the foveal ONL, a glial matrix surrounds outer fibers that form an organized triangular array. They maintain this array in the layer of inner segments (not shown). **(I)** Magnified view of the layer of OPL (at top) and cone pedicles surrounded by abundant Müller cell processes. **(J)** Magnified inset shows a synaptic triad on a pedicle. Note the presynaptic ribbons surrounded by synaptic vesicles. n, nuclei; GCL, ganglion cell layer; IPL, inner plexiform layer; INL, inner nuclear layer; OPL, outer plexiform layer; HFL, Henle fiber layer; ONL, outer nuclear layer. Scale bars: panel A-I = 1 μm; panel J = 200 nm.

Single horizontally oriented slices through each retinal layer of the tissue block (Figure 3A) are colorized to highlight distributions of glia and neurons (Figures 3B–J). Müller glia cell bodies in the INL (Figure 3D), including those contacting the index cone, had smooth nuclear membranes surrounded by voluminous intermediate filament-rich cytoplasm. Müller glia cytoplasm filled in among the neurons of the nuclear layers (Figures 3B,J,H). In the OPL (Figures 3E,I), intricate glial processes surrounded neuropil elements and wrapped pedicles. Synaptic specializations in inner and outer plexiform layers were clearly visible, including synaptic triads in cone pedicles (Figure 3J). Microfilaments were clearly visible within the Müller cytoplasm (Krebs and Krebs, 1991; Burris et al., 2002; shown in Figures 3A,F). Because our sample did not include the ELM, we could not observe centrioles and mitochondria localizing to Müller cells in this region (Borwein, 1983; Stone et al., 2008).

**Figure 4:**
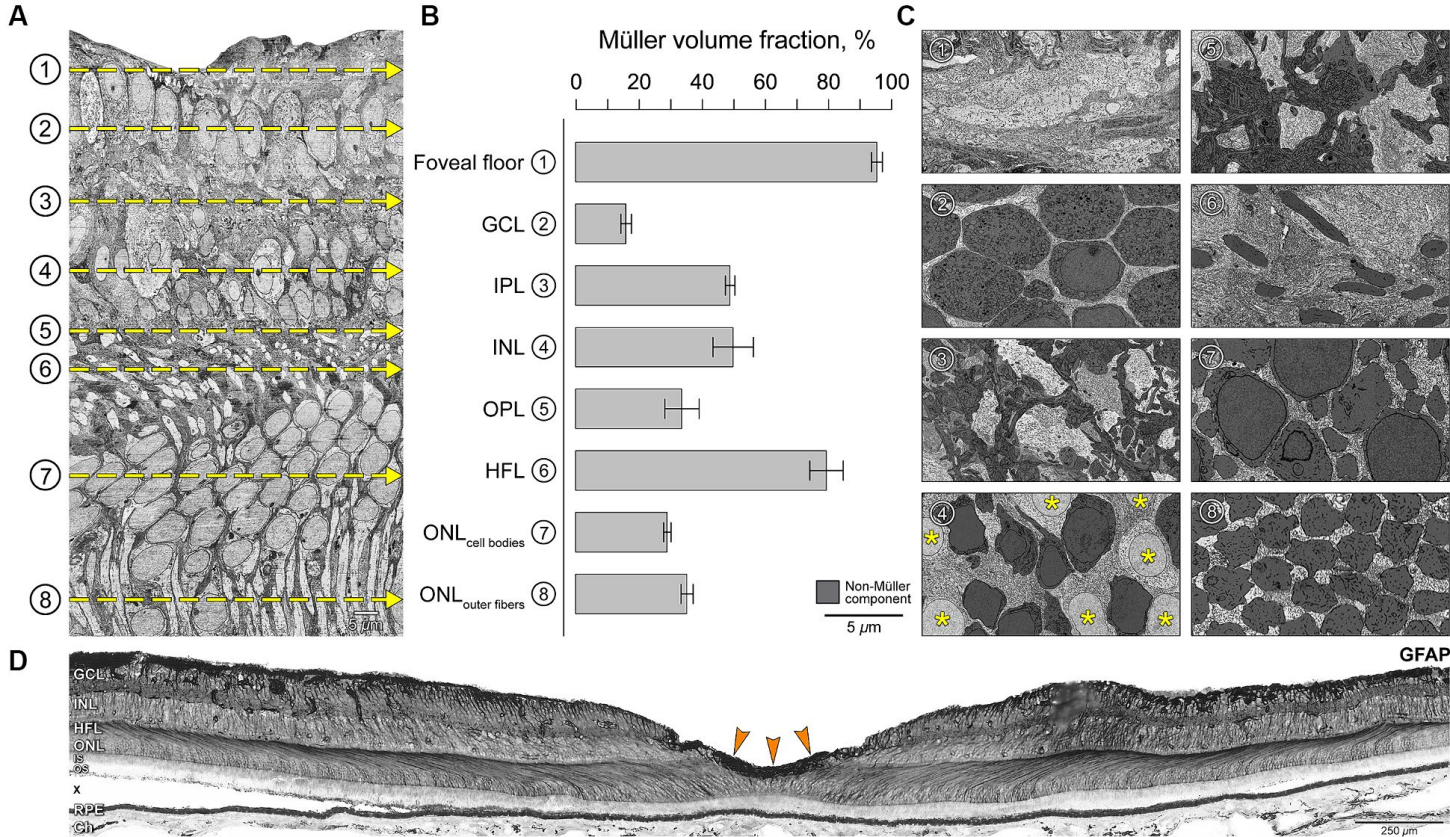
Variations in Müller glia abundance within retinal layers in the foveal center. **(A)** Vertical EM slice is orthogonally reconstructed from horizontal serial sections (Figure 1). **(B)** Four 15 × 15 μm sampling regions from horizontal sections at depths corresponding to the retinal layers (yellow dotted lines in A) were used to calculate the percentage of area occupied by Müller glia. **(C)** Representative horizontal sections demonstrate the neural-glial distribution in corresponding layers. Müller cell somata were located in the INL (asterisk in C4). Non-glial components in each section are shaded dark for identification purposes so that overall lighter panels in C signify greater Müller abundance, corresponding to longer bars in panel B. **(D)** Glial fibrillary acidic protein (GFAP) immunostaining of central retina of a human eye. Chromogenic immunohistochemistry performed in a macula section of a different eye (an 80-year-old female) from the index case. Staining suggests labeling of astrocytes and Müller glia. Note the prominent zone of staining in the inner fovea, corresponding to the bar representing the foveal floor (B1) and suggesting presence of Müller glial mass. The HFL is discernable due to GFAP staining represented by Müller cell trunks. GCL, ganglion cell layer; IPL, inner plexiform layer; INL, inner nuclear layer; OPL, outer plexiform layer; HFL, Henle fiber layer; ONL, outer nuclear layer. Graph data are represented as mean ± standard deviation. *Scale bars*: panel A, C1-8 = 5 μm; panel D = 250 *μ*m.

In the foveal center, we observed striking variation in glial cytoplasm abundance among retinal layers (Figure 4). In individual layers viewed in the tangential plane (Figure 4A), neurons are shaded dark so that overall lighter panels in Figure 4C signify greater Müller abundance. Müller cell somata were in the INL (asterisk in Figure 4C). The percentage of the retinal volume occupied by glia versus non-glia was highest at the foveal floor (95%) and the HFL (80%) (shown as a histogram in Figure 4B). We independently verified this laminar distribution by showing immunoreactivity for GFAP in different eyes (Figure 4D). A prominent staining on the foveal floor suggests the presence of the glial mass (Figure 4D, orange arrows). The HFL is discernable due to GFAP immunoreactivity of Müller cell trunks (Figure 4D).

### Three-dimensional reconstructions

**Figure 5:**
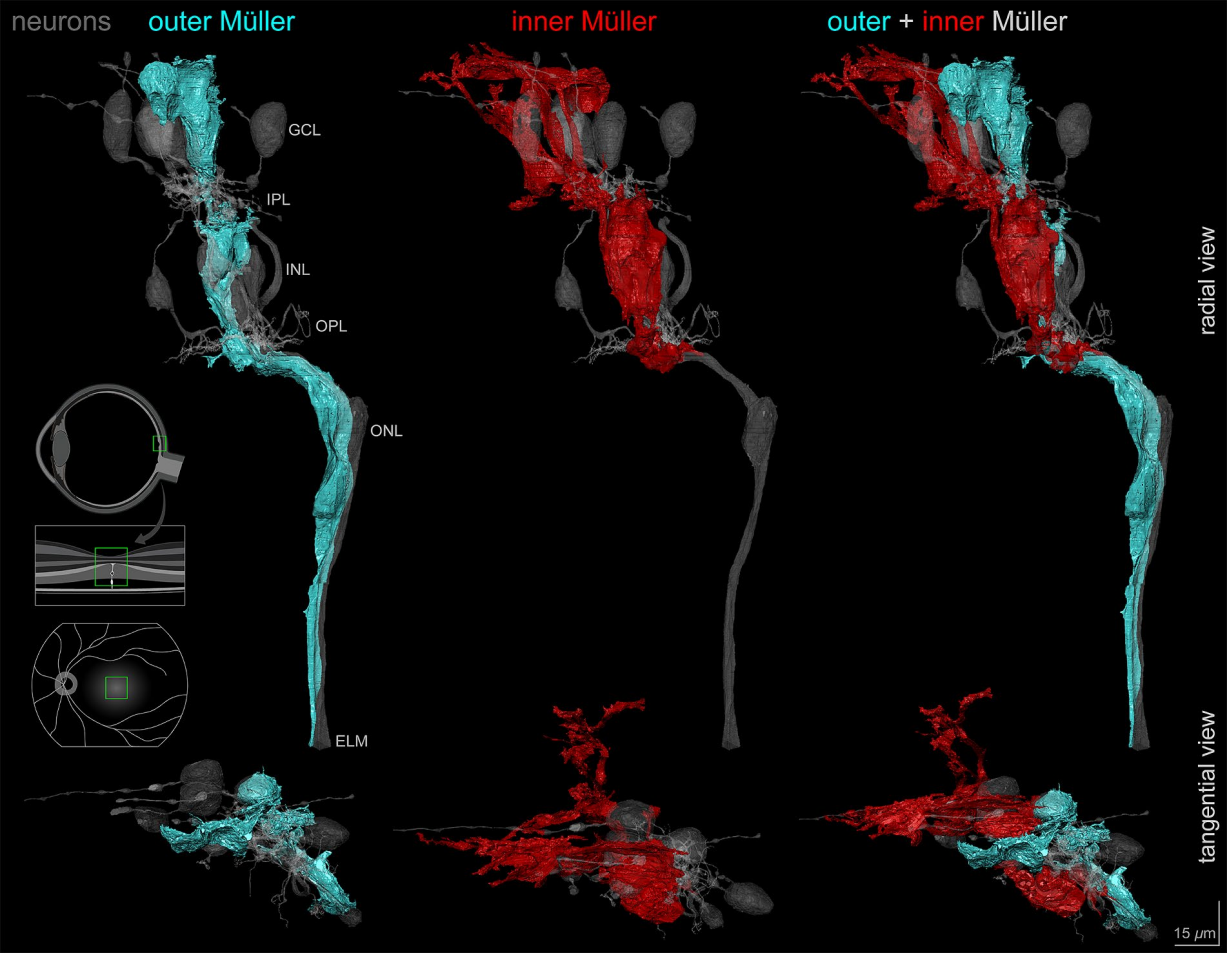
Outer and inner Müller cells in relationship to retinal neurons. Vertical and horizontal views of the outer and inner Müller glia reconstructions demonstrating morphological differences in relation to retinal neurons including the central-most foveal cone (gray). Note the differences in layer extent between the outer (cyan) and inner (red) cell types. Scale bar = 15 μm.

During the reconstruction of Müller glia, we discovered that some cells did not show the expected morphology. Outer Müller glia are the classically defined cells that extend from ILM to ELM. In this block that did not reach the ELM, it was nevertheless possible to observe that the outer Müller cells partially wrapped cone axons along their length (Figure 5), as noted previously (Polyak, 1957; Perry and Cowey, 1988). In contrast, a population of cells extending only from the ILM to the OPL was also identified (Figure 5). These cells characteristically terminated by wrapping around cone pedicles and telodendria (Supplementary Video S2). We classified Müller glia into inner and outer cells based on the vertical extent of trunks heading internally or externally from cell bodies in the INL using serial horizontal sections. We then completely reconstructed one outer and one inner Müller cell contacting the index cone (Figure 5, Supplementary Video S2).

Of interest was the size, complexity, and vertical compartmentalization of individual glia, best seen in a video tour of the reconstructions (Supplementary Video S2; timestamps are in seconds). Both cells had ‘pericellular baskets’ (Polyak, 1941) in ONL-INL-GCL for outer cells and in INL-GCL only for inner cells (9:22, 33:55). These concave depressions consist of a thin layer of cytoplasm bounded by closely spaced plasma membranes and shaped to fit the cell bodies of adjacent neurons. In the GCL, outer Müller cell pericellular baskets completely enveloped a ganglion cell body and contacted the partial surfaces of multiple others. The inner Müller cells covered partial surfaces. Both Müller cell types had numerous small side processes in the plexiform layers (30:32). Abundant lateral processes in the IPL were traced from the outer wall of the glial trunk using 3D skeletons. For 19 processes, the median length was 9.9 μm (range 3.7 to 47.7 μm). Both inner and outer Müller cells inserted finger-like processes into the ILM (55:61) (Syrbe et al., 2017). Neither inner nor outer cells had prominent end feet in this specimen.

### Spatial distribution and morphometric analysis

**Figure 6:**
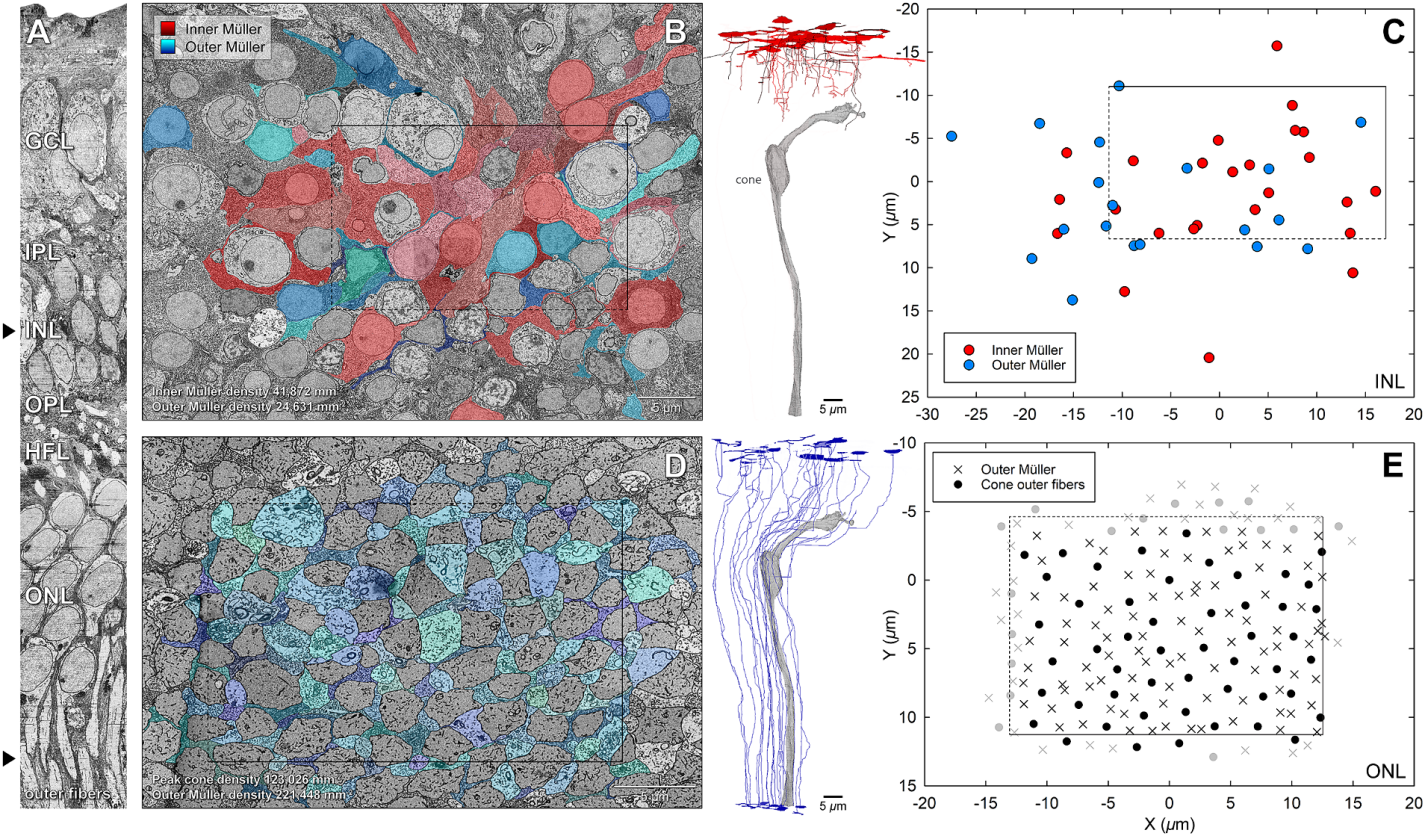
Spatial distribution of Müller cell types in the INL and ONL. Arrowheads in the orthogonal vertical section (left panel) indicate horizontal sections A and C sampled from respective layers. **(A)** Color overlays in the representative tangential INL section designate distinct Müller subtypes, which are determined using morphological criteria and analyzed by serial sections (see Methods). **(B)** Plot shows the distribution of centers of the Müller nuclei. **(C)** Each color overlay in the representative tangential ONL section denotes a separate Müller fiber surrounded by cone outer fibers identified using serial sections. **(D)** Plot shows the distribution of corresponding cones and Müller cells (lower panel). The dotted lines indicate excluded sides and gray data points indicate excluded cells relevant to morphometric analyses. Scale bars = 5 μm.

In addition to complete reconstructions, we skeletonized inner and outer Müller cells for quantitative analysis. Quantification was possible because Müller cell bodies in the INL were reliably more spherical than those of the neurons. Furthermore, inner Müller cell bodies in the INL were located slightly internal to those of the outer Müller cells. Three-dimensional visualization of skeletonized glia demonstrates tight packing and differing extents of inner and outer cells (Supplementary Video S3). The inner Müller cell was recognizable by long, radially arranged beams projecting toward and through the inner retina. Inner Müller cells terminated at the OPL, as shown in red skeletons (Figure 6). Near the ILM in the foveal floor, inner and outer Müller cells both contributed to the glial mass (Figure 2C).

Peak cone density was determined in the ONL near the level of the ELM, where cone outer fibers can be visualized in a single plane without overlap. The measured peak of 123,026 cells/ mm^2^ was at the lower end of the range published for presumed normal eyes of human donors and patients (Curcio et al., 1990; Zhang et al., 2015). In the ONL (Figure 6D), Müller glia trunks occupy space between cone outer fibers. Thus, in the region of peak cone density, the density of outer Müller cells (Figure 6D) was 221,448 cells/ mm^2^, i.e., 1.8 times more numerous than cones (Figure 6E). In this layer, approximately four Müller cells contact each cone outer fiber in the ONL (*n* = 60, cone: outer Müller = 1: 4.2).

To compare the abundance of the two glial cell types, we determined the spatial distribution of inner Müller cells (Figure 6B) in the INL, where they were recognizable by distinctive nuclei surrounded by glial cytoplasm, and unidirectionality of vertical processes directed inwardly. Keeping the same vertically aligned sampling area of ONL, we determined the spatial distribution of outer Müller cell processes in the INL (Figure 6C). Cell densities for inner and outer Müller cells were 41,872 cells/ mm^2^ and 24,631 cells/ mm^2^, respectively, making inner cells 1.7 times more numerous than outer cells.

### Relationship with foveal neural circuitry

**Figure 7:**
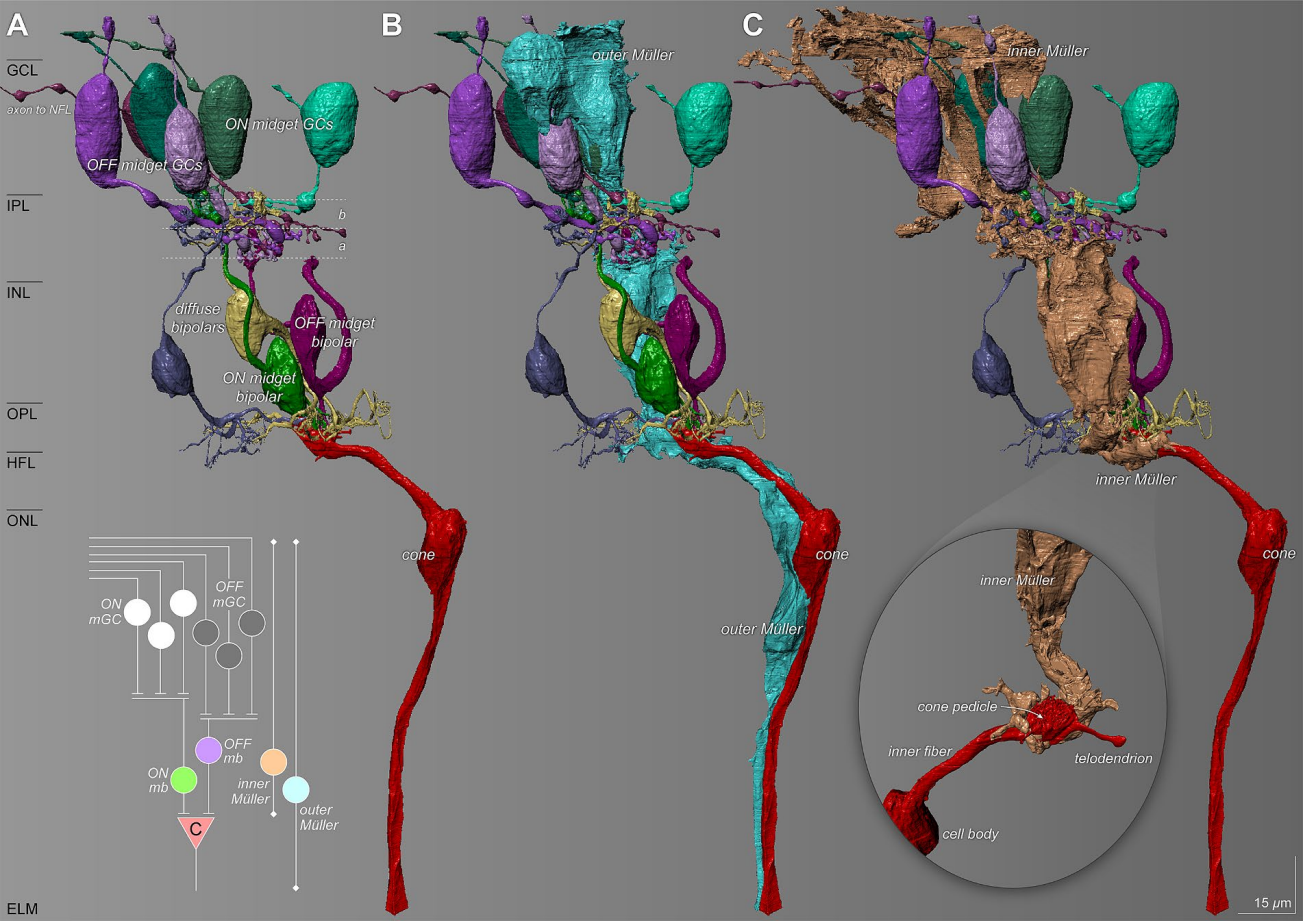
Relationship of Müller glia with the foveal cone-driven midget circuitry. The diagram summarizes ON and OFF circuits from the index cone (c). **(A)** Reconstructed retinal neuron characteristics of the ON and OFF pathways of the midget circuit. a and b refer to the two sublayers of the IPL, corresponding to the branching of the terminals of cone bipolar cells and dendrites of OFF-and ON-center ganglion cells, respectively (Famiglietti and Kolb, 1976). **(B)** An outer Müller cell (teal) wraps around multiple cell bodies and traverses through the synaptic layers spanning the entire circuit. **(C)** An inner Müller cell selectively wraps the cone pedicle (inset) and ensheathes the post-receptoral neurons of the cone-driven circuit. NFL, nerve fiber layer; mGC, midget ganglion cell; mb, midget bipolar; **(C)**, cone. Scale bar = 15 μm.

Müller glia in the foveal center showed an intimate relationship with unmigrated neuronal circuitry that was present in the center of the foveal pit. Figure 7A shows inner and outer Müller cells in relation to reconstructed neurons of cone-driven ON and OFF midget circuits. Other neuron types are present but are not shown here. The outer Müller cell wraps around multiple cell bodies and crosses the synaptic layers, thus spanning the entire circuit, including midget bipolar cells, cone pedicles providing their input, and axons leading to the pedicles (Figure 7B). However, an inner Müller cell selectively wraps only the post-receptoral neurons and the input cone pedicles, without interacting with cone axons in the HFL (Figure 7C). The magnified inset shows how the terminal processes of an inner Müller cell intricately wrap around a cone pedicle.

## Discussion

In this adult human retina, the presence of inner retinal layers in the foveal floor and an undersized avascular zone (Figure 2) supports a family-reported clinical history of a pre-term birth at 28 weeks. Our major finding is a novel class of cells with many characteristics of Müller glia yet do not extend toward the ELM like the classically defined cells. These cells looked exactly like other reconstructed cells, except for lacking an outer process. Features consistent with long-standing descriptions of Müller glia included abundant microfilaments, space-filling between neurons, presence of cell bodies in the INL, side branches, and pericellular baskets. In the human retina, previous visualizations of the foveal center Müller glia have shown only parts of cells (see next paragraph). In various species, individual Müller glia in their entirety have been visualized in Golgi-stains, peroxidase-injection, physical isolation, and artistic conceptions (Polyak, 1941; Robinson and Dreher, 1990; Franze et al., 2007; Bringmann et al., 2018). Using volume electron microscopy to fill this gap, we could distinguish inner cells clearly from those that could be traced through the volume to near the ELM, between cone nuclei, and in parallel with cone outer fibers. One limitation of our study is the lack of the ELM itself in the tissue block. Thus, we could not calculate thickness ratios of the inner and outer retina for direct comparison to clinical studies of pre-term birth retinas. These document persistent inner retinal layers and small FAZ and pit volume (Mintz-Hittner et al., 1999; Bowl et al., 2016, 2018b; Sjöstrand and Popovic, 2020) in childhood up to early adulthood ages (Bowl et al., 2016; Sjöstrand et al., 2017; Sjöstrand and Popovic, 2020) comparable with our donor.

One straightforward interpretation of these data is that inner Müller glia represent a form of arrested development in a subpopulation of cells that were retained in the foveal pit with neurons that did not migrate laterally. All retinal cells develop from progenitor cells in an evolutionarily conserved birth order, in which ganglion cells, cones, and bipolar cells are born in the first half of development, Müller glia and rods in the second half, and amacrine cells in both halves (La Vail et al., 1991; Cepko, 2014). In addition to this temporal sequence, in human and foveate non-human primates, neurons and glia are generated in a spatial sequence starting near the fovea first and then spreading across the periphery (La Vail et al., 1991, Cepko, 2014). Thus, we can surmise that a small number of foveal Müller glia are still being born or being positioned into the INL when post-receptoral neurons have begun lateral migration to form a pit. However, in this specimen, peak cone density was within the normal range (Curcio et al., 1990; Zhang et al., 2015), and Müller glial trunks intertwined with cone cell bodies and outer fibers did not raise suspicions. Thus, we cannot exclude the possibility that inner Müller cells also exist in normal retinas, and their abnormality in this pre-term birth is a lack of migration and not a lack of connection to the ELM.

What is the relation of inner Müller glia to a tissue mass (“Müller cell cone”) in the foveal center in adults of normal term birth? This plug of tissue, shaped like an inverted cone, was revealed by the first electron microscopic examination of human fovea in 1969 and later given its current name (Yamada, 1969; Gass, 1999). Within the foveal floor of presumably normal adults, within a distinct cytoplasmic mass, others have observed nuclei (Hendrickson et al., 2012; Delaunay et al., 2020) or cells negative for the glio-neuronal transmitter recycling enzyme (glutamine synthase) and positive for GFAP (Nishikawa and Tamai, 2001; Delaunay et al., 2020; Reichenbach and Bringmann, 2020). The latter was suggested to represent either astrocytes or Müller glia. Based on our discovery of inner Müller cells among the unmigrated neurons of this preterm birth retina, we hypothesize that the Müller cell cone in a normal term retina represents a small population of glia at the foveal center that has not migrated. In a normal retina, where all foveal neurons have migrated laterally, no neurons or plexiform layers remain for interactions with these Müller cells, which remain relatively undifferentiated. Outside the foveal floor glial mass, both inner and outer Müller cells contact with the ILM (Supplementary Video S2). This tissue layer, comprised of the vitreal aspect of Müller end feet and basal lamina, was very thin in this specimen as it is in a normal fovea (Syrbe et al., 2017).

Cell bodies in the Müller cell cone (Syrbe et al., 2017; Bringmann et al., 2018) were called a second Müller glial type and were not obviously related to our inner Müller glia due to lack of information about connection with ELM. This small group of cells (<35) was considered glia due to a paucity of mitochondria, evenly distributed heterochromatin, and abundant smooth endoplasmic reticulum, compared to the adjacent neurons, and they were inserted into the internal limiting membrane. These foveal floor cells were said to project to the ELM, but data were not provided to support this conclusion due to the use of single section transmission electron microscopy. Thus, it was not possible to determine whether a glial population lacking connections to the ELM was present or absent. Relative to the specimens of Bringmann et al., our foveal specimen differed in several aspects, in addition to visualization techniques and the absence of a pit. Ours also had a thick ONL throughout the tissue volume, scattered pedicles and continuous INL across the foveal floor, and a small umbo in the ganglion cell layer. In contrast, in the Bringmann specimens, all layers internal to the ONL were absent and the ONL itself was thinned in the center [Figure 1 of (Syrbe et al., 2017); Figure 10 of (Bringmann et al., 2018)). Thick and thin ONL configurations have been illustrated previously (respectively (Rochon-Duvigneaud, 1943, Kadomoto et al., 2021) vs. Figure 42 of (Polyak, 1957)] and Figure 8 of (Govetto et al., 2017). These configurations may represent a continuum of developmental lateral migration which also affects cone cell bodies in the ONL or differences in how a tiny foveal center is sampled with single vertical sections. Because lateral migration of neurons creates space for the large pedicles of cone photoreceptors as the smaller inner segments squeeze together toward the foveal center (Schein, 1988; Packer et al., 1990), it has been suggested that delayed inner retinal migration may yield miniature (2.1–4.5) pedicles; these, in turn, may degrade signal transmission (Sjöstrand et al., 2017; Sjöstrand and Popovic, 2020). We did find non-migrated pedicles in the fovea, however, these were typically large (Haverkamp et al., 2000).

In envisioning the foveal center Müller glia of preterm birth in three dimensions, we also estimated their abundance. At the level of the INL, inner cells were 1.7 times more numerous than outer cells (41,872 cells/ mm^2^ vs. 24,631 cells/ mm^2^). Furthermore, outer Müller glia were found to outnumber cones at the level of the cone outer fibers in the ONL by 1.8-fold (221,448 cells/ mm^2^ vs. 123,026 cells/ mm^2^). Because neurons and glia were vertically aligned in this specimen (Supplementary Video S1), a correction for lateral displacement of Henle fibers and post-receptoral cells (Schein, 1988; Drasdo et al., 2007) was unneeded. However, the non-availability of tissue outside the foveal center of our specimen prevented us from defining an eccentricity relationship and comparing it directly with the previous literature. These studies collectively suggest a unity ratio of cones and all Müller glia outside the foveal center in presumably normal human retinas. In macaque monkeys, monoclonal antibodies specific to Müller glia labeled 30,000–37,000 cells/mm^2^ in the parafoveal region compared to 6,000 cells/mm^2^ in the far periphery (Distler and Dreher, 1996). Using serial section electron microscopy and manual reconstruction, Ahmad et al. (2003) found at 575 μm from the foveal center (corresponding to 1°) 26,661 cells/mm^2^ of Müller glia outer trunks and cone pedicles 26,512 cells/mm^2^ for a 1:1 ratio. Using antibodies against glutamine synthetase and differential interference contrast optics to visualize unstained cells, Masri et al. (2021) found in four human specimens that the average peak density of Müller glia at 0.8 mm from the foveal center was 24,000 cells/mm^2^ with high variability. These authors could not assess the foveal floor. They did find that the ratio of Müller glia to cone inner segments in the same retinas within 2 mm eccentricity varied between 1 and 2 glia per cone. Bringmann et al. (2018) counted nuclei in histological cross-sections of an adult human retina and reported 0.73 ± 0.27 cone nuclei per Müller cell nuclei in the foveal center. Of note, foveal Müller cells were described later than the neurons (Polyak, 1941), and their presence, abundance, and distinctive features were debated (Yamada, 1969; Miller and Bernard, 1983; Krebs and Krebs, 1989; Hoang et al., 2002). Some investigators (Delaunay et al., 2020) considered a population of GFAP+ cells in the foveal floor to be astrocytes that may have lost this marker in development (Provis et al., 2000) or perhaps were not detected in specimens lacking the vitreous. The high abundance of foveal Müller glia is novel and should be investigated in other eyes of known developmental history to determine its functional significance.

Our data on layer-specific glial occupancy (Figure 4) are relevant to layer-specific concentrations of macular pigments (lutein and zeaxanthin). These xanthophyll carotenoids are lipophilic nutrients of dietary origin that intercalate into plasma membranes, enhance cone-mediated vision, confer antioxidant protection, and account for the characteristic yellow spot (“macula lutea”) of the human retina (Bone et al., 1988; Grudzinski et al., 2017; Mohn et al., 2017; Kar et al., 2020). Müller glia are major xanthophyll reservoirs as learned through research on glio-degenerative disease (Helb et al., 2008; Powner et al., 2010, 2013; Theelen et al., 2014; Pang et al., 2016; Obana et al., 2017) and persistence of xanthophyll signal after the death of foveal cones (Li et al., 2018; Schultz et al., 2022). An overall high concentration in the foveal center that extends into plexiform and nerve fiber layers (Snodderly et al., 1984; Trieschmann et al., 2008; Li et al., 2020) is parsimoniously accounted for Müller glia, without excluding other cells in those locations. Xanthophylls can be detected chromatographically in prenatal retinal tissue, becoming sufficiently concentrated to permit visibility in the fundus at 6 months of age or later (Bone et al., 1988), presumably as the fovea, and Müller glia within it, mature.

In conclusion, in an adult retina born pre-term, we used volume electron microscopy to discover and characterize a novel class of Müller glia with abundant cytoplasm in the inner retina and lacking an extension toward the ELM-like classically defined cells. Many features of these cells support a hypothesis that Müller glia are impacted by the lateral migration of inner retinal neurons in development, a phenomenon well-known from histology and clinical imaging of children and adults born pre-term (Bowl et al., 2016, 2018a; Sjöstrand et al., 2017). There are insufficient data in this case and in the literature to assess whether a class of foveal Müller glia may lack a connection to the ELM in a normal human retina. More human eyes of different developmental histories are needed to make that determination. Because much cellular-level anatomy is now accessible through OCT-based clinical imaging (Kadomoto et al., 2021; Ramtohul et al., 2022; Gupta et al., 2023), we expect that *in vivo* studies, including longitudinal follow-up (Seely et al., 2022), will allow testing of what aspects of inner Müller glia are unique to pre-term birth.

## Data availability statement

The original contributions presented in the study are included in the article/Supplementary material, further inquiries can be directed to the corresponding author/s.

## Ethics statement

The studies involving humans were approved by Institutional Review Boards at University of Washington and University of Alabama at Birmingham. The studies were conducted in accordance with the local legislation and institutional requirements. The human samples used in this study were acquired from by an IRB-approved protocol, and in collaboration with organ and eye procurement organizations; eye tissue was recovered from a brain-dead donor at the time of organ recovery for transplant. Written informed consent for participation was not required from the participants or the participants’ legal guardians/next of kin in accordance with the national legislation and institutional requirements.

## Author contributions

DK: Visualization, Validation, Methodology, Investigation, Funding acquisition, Formal analysis, Data curation, Conceptualization, Writing – review & editing, Writing – original draft. RaS: Investigation, Writing – original draft. YK: Visualization, Resources, Funding acquisition, Writing – review & editing. OP: Visualization, Resources, Investigation, Data curation, Writing – original draft. RiS: Visualization, Methodology, Writing – original draft. DC: Writing – review & editing, Visualization, Investigation, Data curation, Writing – original draft. KS: Writing – review & editing, Software, Writing – original draft. AP: Visualization, Funding acquisition, Writing – review & editing. DD: Visualization, Supervision, Resources, Project administration, Methodology, Investigation, Funding acquisition, Data curation, Conceptualization, Writing – review & editing, Writing – original draft. CC: Visualization, Supervision, Resources, Project administration, Funding acquisition, Data curation, Conceptualization, Writing – review & editing, Writing – original draft.

## Supplementary material

Supplementary Video S1 Retinal layer landmarks and cellular components of the external fovea. Contrast-normalized rendering of serial sections demonstrates cell bodies and fibers of foveal cones. Inner cone fibers form the Henle fiber layer, which is thin at the foveal center. This video is the most externally located and the largest of two image stacks created in two separate sessions, as described in the Methods section. Scale bar = 15 μm.

Supplementary Video S2 Cellular characteristics of inner, outer Müller, and cones demonstrated by three-dimensional connectomic reconstructions. Scene rendering shows detailed associations between the glial cells and the central-most cone, highlighting cellular features and specialized membranes.

Supplementary Video S3 Inner and outer Müller cell populations in relationship to a central foveal cone. Skeletonized tracings of the cells demonstrate the vertical extension of outer cells reaching the ELM while the inner cells terminate in the OPL.
