# Supplementary material for: Unusual morphology of foveal Müller glia in an adult human born pre-term
Source: Front Cell Neurosci. 2024 Jun 27;18:1409405. doi: 10.3389/fncel.2024.1409405 (PMC11236602; doi:10.3389/fncel.2024.1409405)
Supplement: Supplementary file 4 [file Data_Sheet_1.PDF]

Supplementary Material – Methods for immunohistochemistry

Kar, D., Singireddy, R. R., Kim, Y. J., Packer, O., Schalek, R., Cao, D., Sloan, K. R., Pollreisz, A., Dacey, D. M., Curcio, C. A. Unusual morphology of foveal Muller glia in an adult human born pre-term. Frontiers in Cellular Neuroscience.

More information is available in (Cao et al., 2021, Chen et al., 2022, Anderson et al., 2023)

Ultrastructural findings were compared to immunoreactivity of glial fibrillary acidic protein (GFAP) a marker for astrocytes and Müller glia in human retina (Bringmann et al., 2018). Whole eyes were obtained from deceased human donors (≥ 80 years of age, white, non-diabetic, and ≤6 hours death-to-preservation) to the Advancing Sight Network (Birmingham AL USA.) Eyes were preserved in 4% buffered paraformaldehyde and screened for pathology using *ex vivo* multimodal imaging (Messinger et al., 2023).

From nine eyes with unremarkable central retinas were used for this study, 5x8 mm rectangles of full-thickness eye wall including fovea and optic nerve were dissected. To prevent ice crystals, tissue was cryoprotected in ascending concentrations of sucrose in phosphate buffered saline (PBS) then ascending concentrations of tissue embedding medium and sucrose buffer (HistoPrep, Thermo Fisher Scientific, #SH75-1250D, Rockford IL USA; 4 parts sucrose buffer and 1 part medium then 2:1). Tissue was placed into a cryomold (Electron Microscopy Sciences, #70176-10, Fort Washington PA USA) with embedding medium, oriented, frozen in liquid nitrogen, and stored at -80°C. Tissues were sectioned at 12 µm thickness (Leica CM3050 Vashaw Scientific, Norcross GA USA), air dried at 37°C overnight, and stored at -80°C until used. To confirm normal structure, cryosections were stained with periodic acid Schiff hematoxylin (Vogt et al., 2011).

Sections were processed with a peroxidase immunohistochemistry detection system. Cryosections were warmed, hydrated in PBS at room temperature, and processed for 15 minutes under steam with an antigen retrieval solution (Vector Labs, Cat# H-3300-250) in

glycerol. Endogenous hydrogen peroxide was quenched with 3% H_2O_2 in distilled water (Thermo Fisher Scientific, Cat# H-325-100). Non-specific binding was blocked with 5% mouse serum (Thermo Fisher Scientific, Cat# OB0050) for 1 hour at room temperature, then incubated with 1:500 Polyclonal Rabbit Anti-Glial Fibrillary Acidic Protein (Agilent Technologies, Cat# Z033429-2) in 1xPBS blocking buffer (Thermo Fisher Scientific, Cat# 37525) with 1.5% mouse serum overnight at 4°C. The following day, sections were washed with 1x PBS for 5 minutes 3x, then incubated for 2 hours at room temperature in 1:100 mouse Anti-Rabbit HRP conjugated secondary antibody (Santa Cruz, Cat# sc-2457) in 1xPBS blocking buffer (Thermo Fisher Scientific, Cat# 37525) with 1.5% mouse serum. Subsequently, sections were washed in 1x PBS for 5 minutes x2 and washed with dH_2O for 5 minutes x2, then a purple color developed with ImmPACT Peroxidase Substrate (Vector Labs, Cat# SK-4605) for 2-15 minutes under visual control. After washing, sections were counterstained with hematoxylin (Poly Scientific R&D Corp, Cat# K047). Coverslips were mounted with aqueous medium (VectaMount AQ, Vector Labs, Cat# H-5501-60), sealed with nail polish, air-dried, and stored at 4ºC. Negative control slides omitted primary antibody. Glass slides were scanned using a 20X objective and a robotic microscope stage (Olympus VSI 120, CellSens; Olympus, Center Valley PA).

ANDERSON, D. M. G., KOTNALA, A., MIGAS, L., PATTERSON, N. H., TIDEMAN, L., CAO, D., ADHIKARI, B., MESSINGER, J. D., ACH, T., TORTORELLA, S., VAN DE PLAS, R., CURCIO, C. A. & SCHEY, K. L. 2023. Lysolipids are prominent in subretinal drusenoid deposits, a high-risk phenotype in age-related macular degeneration. *Frontiers in Ophthalmology,* 3**,** 1258734.

BRINGMANN, A., SYRBE, S., GORNER, K., KACZA, J., FRANCKE, M., WIEDEMANN, P. & REICHENBACH, A. 2018. The primate fovea: Structure, function and development. *Prog Retin Eye Res,* 66**,** 49-84.

CAO, D., LEONG, B., MESSINGER, J. D., KAR, D., ACH, T., YANNUZZI, L. A., FREUND, K. B. & CURCIO, C. A. 2021. Hyperreflective foci, OCT progression indicators in age-related macular degeneration, include transdifferentiated retinal pigment epithelium. *Invest Ophthalmol Vis Sci,* 62**,** 34.

CHEN, L., CAO, D., MESSINGER, J. D., ACH, T., FERRARA, D., FREUND, K. B. & CURCIO, C. A. 2022. Histology and clinical imaging lifecycle of black pigment in fibrosis secondary to neovascular age-related macular degeneration. *Exp Eye Res,* 214**,** 108882.

MESSINGER, J. D., BRINKMANN, M., KIMBLE, J. A., BERLIN, A., FREUND, K. B., GROSSMAN, G. H., ACH, T. & CURCIO, C. A. 2023. Ex vivo OCT-based multimodal imaging of human donor eyes for research in age-related macular degeneration. *J Vis Exp*.

VOGT, S. D., CURCIO, C. A., WANG, L., LI, C.-M., MCGWIN, G., JR, MEDEIROS, N. E., PHILP, N. J., KIMBLE, J. A. & READ, R. W. 2011. Retinal pigment epithelial expression of complement regulator CD46 is altered early in the course of geographic atrophy. *Exp Eye Res,* 93**,** 413-423
